# Physical activity and social adaptation in high school students: the chain mediating roles of interpersonal relationships and psychological resilience

**DOI:** 10.3389/fpubh.2026.1740134

**Published:** 2026-03-11

**Authors:** Yifan Chen, Jingyu Ma, Jinkun Li, Yu Zang

**Affiliations:** 1 School of Physical Education and Sports, Central China Normal University, Wuhan, China; 2 College of Life Sciences, Wuhan University, Wuhan, China; 3 Xuchang Senior High School, Xuchang, China

**Keywords:** physical activity, social adaptation, interpersonal relationship, psychological resilience, adolescents

## Abstract

**Objective:**

This study was designed to test the relationship between physical activity and social adaptation among high school students, as well as the mediating effects of interpersonal relationships and psychological resilience in this association.

**Methods:**

High school students (*N* = 1,093) in Henan Province, China, completed the International Physical Activity Questionnaire-Short From (IPAQ-S), the Interpersonal Relationship Comprehensive Diagnostic Scale, the Adolescent Psychological Resilience Scale, and the Social Adaptive Capacity Diagnostic Scale in their classrooms. Data analysis was performed using SPSS software and the Process plugin, and data visualization was carried out using RStudio.

**Results:**

(1) Physical activity was correlated with social adaptation, as well as with both mediators. (2) Interpersonal relationships and psychological resilience independently mediated the relationship between physical activity and social adaptation. Furthermore, physical activity was associated with social adaptation through the chain mediation effects of interpersonal relationships and psychological resilience.

**Conclusion:**

The results highlight interpersonal relationships and psychological resilience as mechanisms of the association between high schoolers’ physical activity and social adaptation. These findings provide a practical basis for developing interventions aimed at improving high school students’ social adaptation abilities.

## 1 Introduction

The high prevalence of health problems among adolescents poses a major threat to global health in the 21st century (1). In 2020, the Ministry of Education of China jointly issued the Opinions on Deepening the Integration of Sports and Education to Promote the Healthy Development of Adolescents, which pointed out that we should establish the concept of health first (2). There is a global consensus that adolescents can benefit across multiple health domains by engaging in regular, moderate- to vigorous-intensity physical activity daily (3, 4). Physical activity yields associated with physical, psychological, and interpersonal relationships of health. It moderately alleviates anxiety symptoms (5), reduces non-communicable diseases (6), and improvements in cardiovascular health were positively associated with improvements in mental well-being (7–9). Physical activity has been shown to be strongly associated with building peer relationships and fostering positive interpersonal relationships (10, 11).

In addition to its physical, psychological, and interpersonal relationships, health includes the capacity for social adaptation (12). Physical activity has been shown to be positively associated with this component of health (13–15). Current challenges in social adaptation faced by high school students manifest as social anxiety, declining academic performance, emotional problems, poor teacher-student communication, and identity confusion (16, 17). These problems in social adaptation can lead to depression and difficulty adapting to collective life, severely impairing future development.

High school students face significant social challenges and are prone to various psychological and behavioral problems related to low social adaptation (18, 19). However, research on the underlying mechanisms linking physical activity and social adaptation in adolescence remains limited (20). Given the current health status of high school students, there is an important need to explore the mechanisms of social adaptation among high school students to foster their adaptive development.

**Figure 1:**
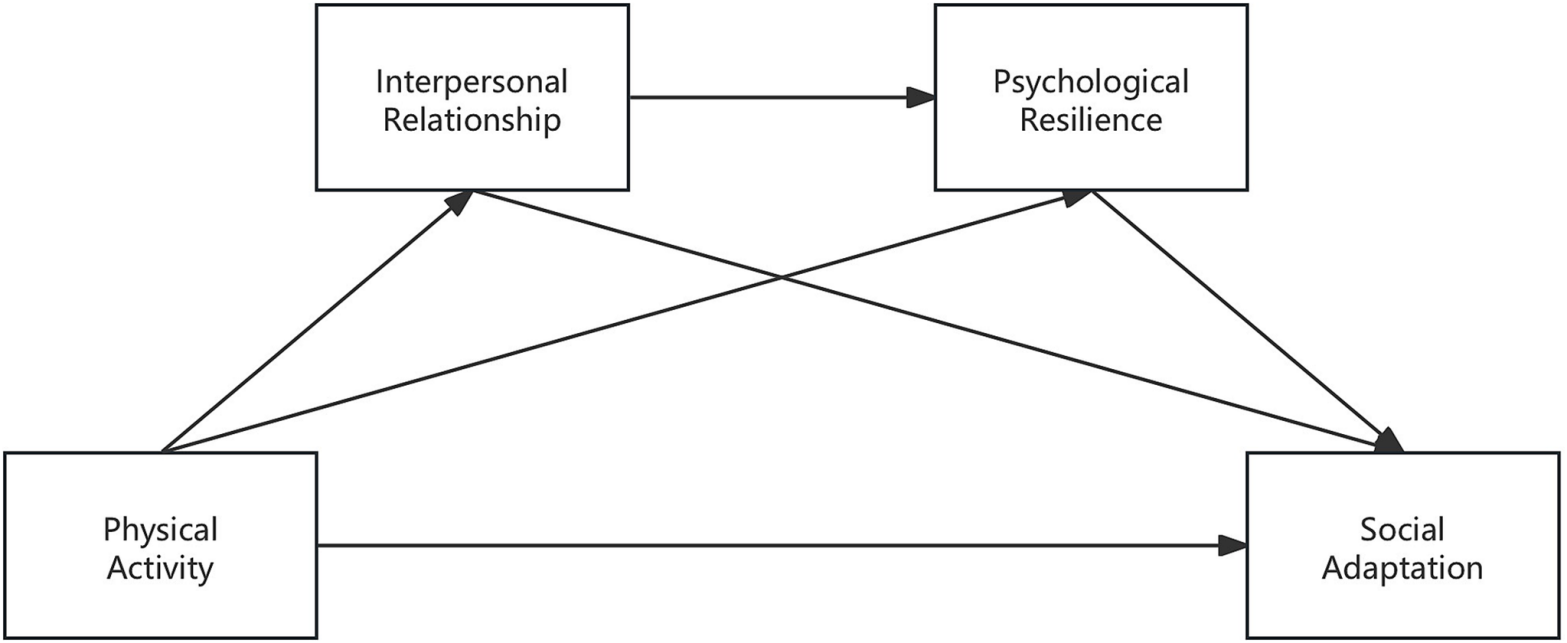
Relationships among the concepts of the study.

This study positions physical activity as a health promotion strategy that is associated with higher social adaptation by way of two psychological variables—interpersonal relationships and psychological resilience—as mediators. There is evidence that these potential mediators are correlated with both physical activity and with social adaptation, and with each other, allowing a meaningful test of a mediation model. See Figure 1. The results will have implications fooptimizing social adaptation among high school students, provide empirically based recommendations, and expand the theoretical understanding of the positive psychological effects of physical activity in this adolescent population.

### 1.1 The relationship between physical activity and social adaptation

As a non-pharmacological, low-cost health strategy favored by many, physical activity has been extensively studied in recent years (21, 22). Physical activity provides adolescents with a platform for social interaction and interpersonal engagement, allowing them to communicate emotions, share joy, establish broader interpersonal networks, and enhance their social adaptation capacities (23). Thus, adolescents who participate in physical activity may have an impact on their social adaptability (24). An empirical study of 3,176 adolescents found that physical activity appears to influence social adaptation, with boys showing influence in moderate-intensity and vigorous-intensity physical activities, while girls may only demonstrate an influence on moderate-intensity physical activities (25). A study involving 980 teenagers indicates that those who engage in physical activities, particularly team-based activities, may influence on their social adaptation (26). Physical activity is considered a precursor variable of social adaptation (27), significantly and positively predicting an adolescent’s social adaptation ability (28). The current study further explores the relationship between physical activity and social adaptation among high school students. Hypothesis 1 is proposed: Physical activity will be significantly and positively associated with social adaptation behavior in high school students.

### 1.2 Psychological mediators of the link between physical activity and social adaptation

Social adaptation theory posits that individuals can progressively achieve socialization through mutual adaptation between themselves and the social environment (29). The theory holds that physical and mental strength, along with self-restraint, directly influence this process. A study of 2,100 adolescents found that individuals with more interpersonal conflicts may experience higher emotional stress, thereby reducing their coping and adaptability (30). Highly resilient individuals are more likely to maintain positive attitudes in social environments, making them more adaptable (31). One study of a high-risk sample found that psychological resilience promoted social adaptation capacities (32). Therefore, interpersonal relationships and psychological resilience are introduced as mediating variables to test a chain mediation model.

#### 1.2.1 The mediating role of interpersonal relationships

Self-efficacy theory suggests that physical activity enhances self-efficacy (i.e., confidence) (33). Individuals with high self-efficacy tend to be more confident in social interactions, facilitating the establishment of positive interpersonal relationships and subsequently elevating their social adaptation (34). In 1977, psychiatrist G. L. Engel proposed the biopsychosocial model, emphasizing that biological, psychological, and social factors do not exist in isolation but form an interactive, dynamic system (35). Adequate physical activity serves as the physiological foundation for coping with social challenges. On the other hand, Social adaptability may limit adolescents’ social participation (36) and indirectly damage interpersonal relationships by influencing their emotions. Physical activity is thought to enhance adolescents’ social and emotional skills, improve their positive emotional states, and directly or indirectly ameliorate their interpersonal skills (37).

Several studies have provided evidence of this view. One study indicated that 60–70 min of moderate-intensity physical activity per week significantly reduced depressive symptoms and improved adolescents’ relationships with friends, teachers, and parents (38). Active participation in team-based physical activities can effectively expand an adolescent’s social network and provide profound emotional satisfaction (39). Engaging in group physical activities can foster new friendships and promote positive peer relationships (40), thereby enhancing interpersonal skills. As a behavior rich in emotional experiences, physical activity strengthens adolescents’ emotional connection abilities by establishing and improving social networks (41). During physical activity, adolescents improve their social skills, teamwork abilities and emotional regulation skills, enabling them to better adapt to environmental changes, reduce feelings of loneliness and enhance their social adaptation capacities (42). Therefore, regular physical activity can optimize adolescents’ interpersonal relationships, thereby improving their level of social adaptation and reducing behavioral problems. High social adaptation requires individuals to find satisfaction and happiness in their social environment. An optimal state necessitates health across physical activity, interpersonal relationships, and social adaptation. Given this evidence, Hypothesis 2 is proposed: Interpersonal relationships will mediate the relationship between physical activity and social adaptation among high school students.

#### 1.2.2 The mediating role of psychological resilience

Psychological resilience, also referred to as the capacity to confront adversity (43), is not a static personal trait but a dynamic process involving adaptation, recovery, and growth in response to stress (44). It denotes the dynamic process through which individuals maintain positive adaptation when facing significant adversity (45). Psychological resilience possesses certain trait-like characteristics, functioning as a developable resource influenced by both personal traits and environmental factors, manifesting as the capacity to rapidly adjust one’s state when encountering adversity (46).

The broaden-and-build theory of positive emotions that positive emotions, as short-term experiences, function to broaden individuals’ cognitive-behavioral repertoires, which in turn build enduring personal resources over the long term (47). Research indicates that psychological resilience is a core psychological ability in an individual’s developmental journey, enabling them to exhibit positive developmental trajectories, demonstrate effective adaptive processes, and display sound social adaptation capacities even when confronted with life difficulties, major disasters, or adversity (48, 49). This process highlights the dynamic interplay between protective factors and external threats (45).

A study involving middle school students found that participation in organized physical activity enhanced positive cognitive reappraisal, thereby fostering characteristics of resilience such as tolerance for frustration and promoting proactive coping abilities (50). In a meta-analysis, it was concluded that physical activity has a significant effect on interventions for reducing anxiety and enhancing self-esteem among adolescents, indirectly shaping self-perception (51). Individuals with strong psychological resilience cope better with stress, exerting a significant positive effect on the development of social adaptation (32). Based on this, Hypothesis 3 is proposed: Psychological resilience will mediate the relationship between physical activity and social adaptation among high school students.

#### 1.2.3 The chain mediating roles of interpersonal relationships and psychological resilience

There is a connection between close friendship and psychological resilience in interacting with the social environment. Supportive friendships may promote the development of psychological resilience among teenagers (52). Furthermore, adolescents with high psychological resilience levels should have good interpersonal relationship (53). A study focusing on late adolescents found that those with higher psychological resilience better counteracted the detrimental effects of interpersonal ruptures on well-being. This suggests that positive interpersonal relationships are a significant source of psychological resilience (54). Accordingly, Hypothesis 4 is proposed: Interpersonal relationships and psychological resilience will sequentially mediate the association between physical activity and social adaptation behavior in high school students.

## 2 Materials and methods

### 2.1 Participants

Using the two stage random sampling method, we first randomly selected three schools from the overall sampling framework of schools in different administrative regions of Henan Province in Central China, including public and private high schools. Subsequently, student samples were randomly selected from multiple grades and classes of the three chosen schools. Eventually, a total of 1,093 high school students were invited to fill out the questionnaire. Questionnaires will be distributed offline and collected uniformly within 30 min. Participants were informed that these data would only be used for research purposes, and the data were anonymous. All students voluntarily decided whether to participate or not. The data cleaning procedure involved the exclusion of questionnaires that were blank, incomplete, or exhibited uniform responses. There were 953 valid questionnaires were obtained, with a validity rate of 87.19%. Among the 953 high school students, 580 were male (60.9%) and 373 were female (39.1%); ages ranged from 14 to 18 years (M = 15.91, SD = 0.74); 444 were in Grade 10 (46.6%), 502 in Grade 11 (52.7%), and 7 in Grade 12 (0.7%); 176 were only children (18.5%), while 777 had at least one sibling (81.5%); 519 were from urban areas (54.5%), 177 from towns (18.6%), and 257 from rural areas (27.0%); 676 experience in student government (70.9%), and 277 had no such experience (29.1%). Ethical approval for the study was granted by the researcher’s university.

### 2.2 Measurement

#### 2.2.1 Physical activity

Physical activity, assessed using the International Physical Activity Questionnaire-Short Form (IPAQ-S) (55), was calculated as the total amount of energy spent in activities with different metabolic equivalents (MET-min). 1 MET is the resting metabolic rate, equivalent to the oxygen consumption while sitting quietly in a chair (approximately 3.5 mL/kg/min). An activity with an intensity of 2 METs consumes twice the energy of sitting at rest. The IPAQ-S covers walking (MET value 3.3), moderate-intensity physical activities (MET value 4.0), and vigorous-intensity physical activities (MET value 8.0). The questionnaire asks about the frequency and time spent in activities at each intensity level. The MET-min score was calculated to represent the total energy expended on physical activity for one week. Three categories are created based on the activity intensity, activity frequency, and MET-min score (see Table 1 for criteria). This questionnaire is widely used internationally and has shown good reliability and validity (56, 57).

**Table 1:** Group criteria based on physical activity intensity and frequency reported on the IPAQ-S.

Group	Criteria (meeting any one)
High physical activity group	1. Vigorous-intensity activity ≥3 days/week AND total MET-min/week >1,500
	2. Combined walking, moderate, or vigorous activity MET-min/week ≥3,000
Moderate physical activity group	1. ≥3 days of vigorous activity of ≥20 min/day
	2. ≥5 days of moderate-intensity activity or walking of ≥30 min/day
	3. ≥5 days of any combination of walking, moderate, or vigorous activity achieving ≥600 MET-min/week
Low physical activity group	Does not meet criteria for High or Moderate physical activity groups

#### 2.2.2 Interpersonal relationships

Interpersonal relationships were assessed using the Interpersonal Relationship Comprehensive Diagnostic Scale (58). The measure is widely used among Chinese adolescents and has shown good reliability and validity (59). This 28-item scale covers four dimensions: communication difficulties, socializing and friendship difficulties, responding to others’ difficulties, and heterosocial difficulties. Respondents answer “yes” (1 point) or “no” (0 points). Item scores on each subscale are summed, with a subscale score > 3 indicating problems in that area. The sum of all items is reverse scored, ranging from 0 to 28. A total score between 0 and 8 indicates that the individual has no or few troubles in interpersonal relationships, is skilled at interacting with others, leads a fulfilling and colorful life, is likely to win others’ favor, and has good interpersonal relationships. A score between 9 and 14 indicates that the individual has some minor troubles in interpersonal relationships. Their popularity is average, and their relationships with others fluctuate, sometimes positive and sometimes negative. A score between 15 and 28 indicates that the individual has serious problems in interpersonal relationships. They may not be skilled at communication, be introverted, or show obviously offensive behaviors. The Cronbach’s *α* coefficient for the scale in this study was 0.824.

#### 2.2.3 Psychological resilience

Psychological resilience was measured using the Adolescent Psychological Resilience Scale (60). The measure designed for adolescents. This 27-item scale has five dimensions: goal focus, interpersonal assistance, family support, emotional control, and positive cognition. It uses a 5-point Likert scale, with 1 = completely inconsistent and 5 = completely consistent. Items 1, 2, 5, 6, 9, 12, 15, 16, 17, 21, 26, 27 are reverse-scored. A higher total sum indicates better psychological resilience. The Cronbach’s *α* coefficient for the scale in this study was 0.811.

#### 2.2.4 Social adaptation

Social adaptation was evaluated using the Social Adaptive Capacity Diagnostic Scale (58). The measure is widely used among Chinese adolescents and has shown good reliability and validity (61). This 20-item scale measures five dimensions: peer relationship skills, self-management skills, learning skills, compliance skills, and expression willingness skills. Odd-numbered items are scored −2 for “Yes,” 0 for “Uncertain,” and +2 for “No”; even-numbered items are reverse-scored. Higher scores indicate higher social adaptation. The Cronbach’s *α* coefficient for the total scale in this study was 0.739.

### 2.3 Statistical analysis

Data were analyzed using SPSS 26.0. The Process 3.5 macro for SPSS was used for mediation analysis with 5,000 bootstrap samples. RStudio 4.4.3 was used for data visualization.

## 3 Results

### 3.1 Test of common method bias

The data were collected using adolescents’ self-report questionnaires, and thus may have been affected by common method bias (62). Therefore, Harman’s single-factor test adopted to examine this possibility. Exploratory factor analysis yielded twenty factors with eigenvalues greater than 1, the first of which explained 12.20% of the variance. This value is lower than the critical value of 40%, so it can be judged that the results were not seriously affected by common method bias.

### 3.2 Descriptive statistics and correlation analysis

**Figure 2:**
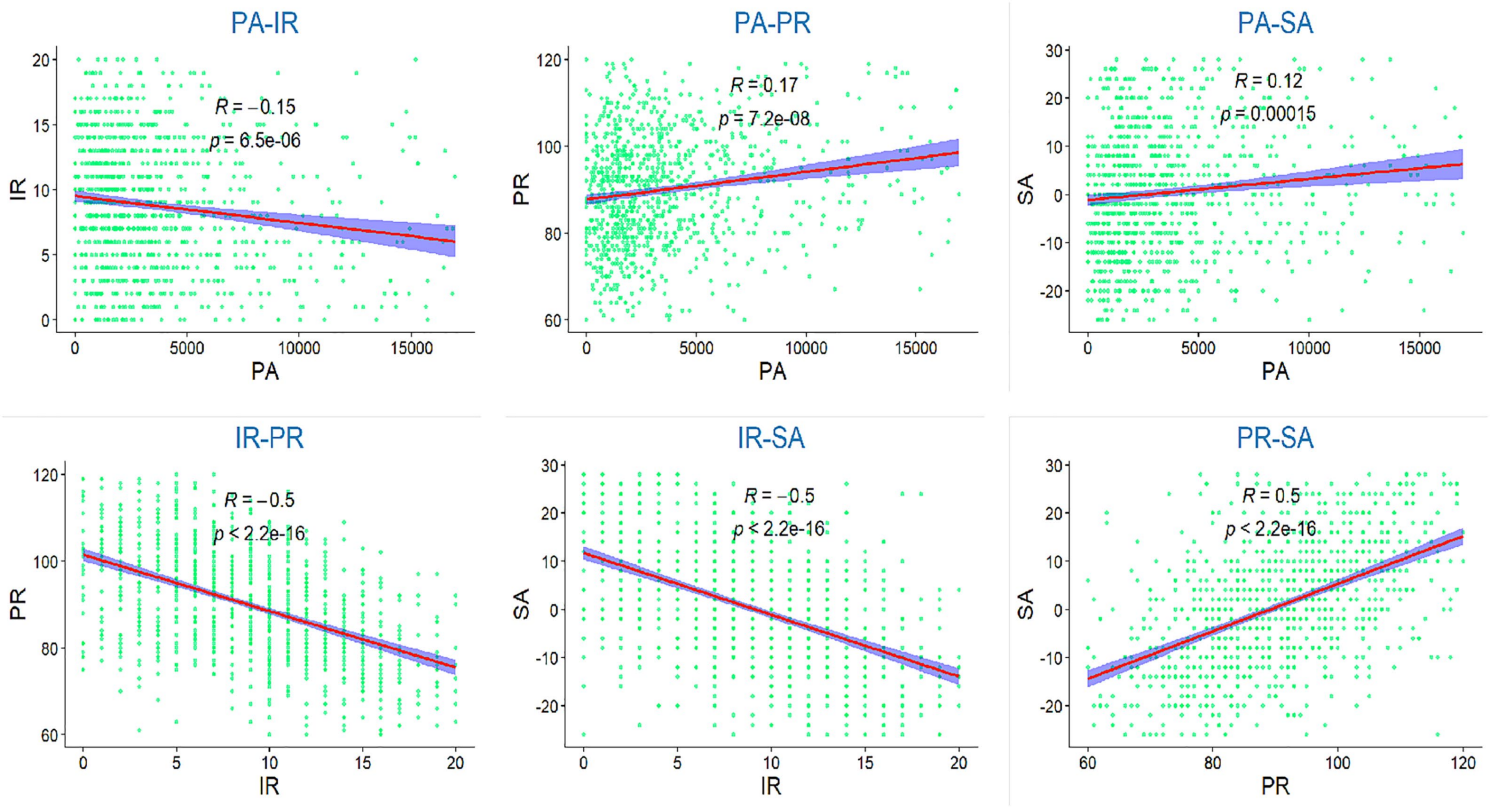
Visualized bivariate correlation among research variables.

Independent samples t tests and one-way ANOVA tests were used to compare demographic groups on the study variables. Table 2 shows that there were significant differences in physical activity by grade (*p* < 0.001). Grade 1 students had significantly less physical activity than Grade 2 and Grade 3 students. There were significant differences in physical activity (*p* < 0.001) and social adaptation (*p* < 0.001) in terms of gender. Girl had significantly less physical activity and social adaptation than boy. There were significant differences in physical activity in terms of place residence (rural, town, city) (*p* < 0.001). There were significant differences in physical activity (*p* < 0.001) and social adaptation (*p* < 0.05) regarding whether there was experience in student government. Descriptive statistics and bivariate correlation analysis were conducted on the study variables. As shown in Table 3 and Figure 2, physical activity was negatively correlated with interpersonal relationship score (*r* = −0.15, *p* < 0.01), and positively correlated with psychological resilience (*r* = 0.17, *p* < 0.01) and social adaptation (*r* = 0.12, *p* < 0.01). Interpersonal relationship score were negatively correlated with both psychological resilience (*r* = −0.50, *p* < 0.01) and social adaptation (*r* = −0.50, *p* < 0.01), while psychological resilience was positively correlated with social adaptation (*r* = 0.50, *p* < 0.01).

**Table 2:** Sample demographic characteristics.

Variable	Total	Proportion	*t*/*f*-value	PA	IR	PR	SA
	(*N* = 953)	%	*p* value	M ± SD	M ± SD	M ± SD	M ± SD
Grade							
First year	444	46.6		3110.56 ± 3115.59	8.46 ± 4.90	91.22 ± 12.58	0.86 ± 12.71
Second year	502	52.7		4199.51 ± 3717.82	8.99 ± 5.03	89.20 ± 13.26	0.20 ± 12.74
Third year	7	0.7		8879.00 ± 4711.71	10.00 ± 4.69	90.43 ± 13.55	1.43 ± 8.38
			*f*-value	15.74	1.57	2.89	0.34
			*P* value	0.001	0.21	0.06	0.71
Gender							
Male	580	60.9		4511.26 ± 3913.90	8.62 ± 5.17	90.56 ± 13.21	1.74 ± 12.60
Female	373	39.1		2506.34 ± 2343.41	8.96 ± 4.63	89.51 ± 12.59	−1.38 ± 12.63
			*t*-value	9.89	−1.07	1.22	3.72
			*P* value	0.001	0.28	0.223	0.001
Only child							
Yes	176	18.5		3582.96 ± 3552.43	8.74 ± 5.07	89.25 ± 13.21	1.52 ± 13.69
No	777	81.5		3759.07 ± 3519.45	8.75 ± 4.95	90.35 ± 12.92	0.29 ± 12.46
			*t*-value	−0.60	−0.02	−1.018	1.16
			*P* value	0.55	0.98	0.31	0.25
Region							
City	519	54.5		3205.93 ± 3056.42	8.57 ± 4.92	90.27 ± 13.08	1.29 ± 12.82
Town	177	18.6		4084.18 ± 3980.29	9.29 ± 4.98	89.79 ± 13.53	−1.08 ± 12.17
Village	257	27.0		4531.59 ± 3886.91	8.74 ± 5.05	90.15 ± 12.42	0.07 ± 12.73
			*f*-value	12.98	1.39	0.09	2.53
			*P* value	0.001	0.25	0.91	0.08
Experience in student government							
Yes	676			3242.64 ± 2985.50	8.89 ± 4.82	90.46 ± 12.95	1.19 ± 12.74
No	277			4907.00 ± 4367.33	8.43 ± 5.30	89.40 ± 13.04	−1.12 ± 12.47
			*t*-value	−5.81	1.25	1.14	2.57
			*P* value	0.001	0.21	0.26	0.01

M, mean; SD, standard deviation; PA, Physical Activity; IR, Interpersonal Relationships (positive relationships; reverse scored); PR, Psychological Resilience; SA, Social Adaptation.

**Table 3:** Descriptive statistics and bivariate correlations (*N* = 953).

Variables	M ± SD	PA	IR	PR	SA
PA	3726.54 ± 3524.35	1			
IR	8.75 ± 4.97	−0.15^**^	1		
PR	90.15 ± 12.98	0.17^**^	−0.50^**^	1	
SA	0.52 ± 12.70	0.12^**^	−0.50^**^	0.50^**^	1

**p < 0.01; M, mean; SD, standard; PA, physical activity; IR, interpersonal relationships; PR, psychological resilience; SA, social adaptation.

### 3.3 Tests of hypotheses

First, we tested the study variables for multicollinearity. VIF values were all less than 10, which can be judged as demonstrating no multicollinearity problem. Second, We input demographic variables as control variables into all analyses.

As shown in Table 4, regression analyses showed that physical activity significantly positively predicted social adaptation (*β* = 0.136, *p* < 0.001). When physical activity was tested as a predictor of interpersonal relationship score, it had a significant negative predictive effect (*β* = −0.164, *p* < 0.001); When physical activity and interpersonal relationship score were tested simultaneously as predictors of psychological resilience, physical activity had a significant positive predictive effect (*β* = 0.130, *p* < 0.001), while interpersonal relationship score had a significant negative predictive effect (*β* = −0.477, *p* < 0.001). When physical activity, interpersonal relationship score and psychological resilience were simultaneously tested as predictors of social adaptation, physical activity had no significant predictive effect on social adaptation, interpersonal relationship score had a significant negative predictive effect (*β* = −0.340, *p* < 0.001), and psychological resilience had a significant positive predictive effect (*β* = 0.324, *p* < 0.001).

**Table 4:** Regression analysis of positive interpersonal relationships (reverse scored) and resilience as chain mediators of the association between physical activity and social adaptation.

Regression equation		Overall fit coefficient			Significance of regression coefficient		95% Confidence interval	
Outcome variable	Predicator variables	*R*	*R* ^2^	*F*	*β*	*t*	LLCI	ULCI
SA	Grade	0.23	0.05	7.28	−0.008	−0.183	−2.3615	1.9593
	Gender				−0.142	−4.114^***^	−5.4660	−1.9353
	Age				−0.065	−1.452	−2.6134	0.3909
	Only child				−0.013	−0.381	−2.5272	1.7059
	Region				−0.067	−2.008^*^	−1.9530	−0.0223
	Class cadre experience				−0.139	−4.135^***^	−5.7250	−2.0396
	PA				0.136	4.013^***^	0.0003	0.0007
IR	Grade	0.18	0.03	4.33	0.092	2.039^*^	0.0333	1.7416
	Gender				0.004	0.110	−0.6588	0.7371
	Age				−0.005	−0.108	−0.6267	0.5611
	Only child				−0.014	−0.405	−1.0094	0.6642
	Region				0.052	1.553	−0.0796	0.6837
	Class cadre experience				−0.022	−0.650	−0.9700	0.4871
	PA				−0.164	−4.773^***^	−0.0003	−0.0001
PR	Grade	0.52	0.27	43.97	−0.030	−0.754	−2.6856	1.1946
	Gender				−0.032	−1.052	−2.4297	0.7341
	Age				−0.057	−1.450	−2.3406	0.3515
	Only child				0.039	1.354	−0.5883	3.2052
	Region				−0.016	−0.553	−1.1102	0.6220
	Class cadre experience				−0.086	−2.928^**^	−4.1155	−0.8124
	PA				0.130	4.330^***^	0.0003	0.0007
	IR				−0.477	−16.889^***^	−1.3899	−1.1005
SA	Grade	0.60	0.37	60.13	0.047	1.280	−0.6174	2.9311
	Gender				−0.130	−4.587^***^	−4.8293	−1.9352
	Age				−0.049	−1.334	−2.0690	0.3948
	Only child				−0.032	−1.180	−2.7796	0.6918
	Region				−0.036	−1.310	−1.3204	0.2635
	Class cadre experience				−0.122	−4.407^***^	−4.9224	−1.8889
	PA				0.013	0.457	−0.0002	0.0002
	IR				−0.340	−11.293^***^	−1.0195	−0.7176
	PR				0.324	10.663^***^	0.2589	0.3757

***p < 0.001, **p < 0.01, *p < 0.05. PA, physical activity; IR, interpersonal relationships; PR, psychological resilience; SA, social adaptation. LLCI, lower limit of the 95% confidence interval (95%CI); ULCI, upper limit of the 95% confidence interval (95%CI).

**Figure 3:**
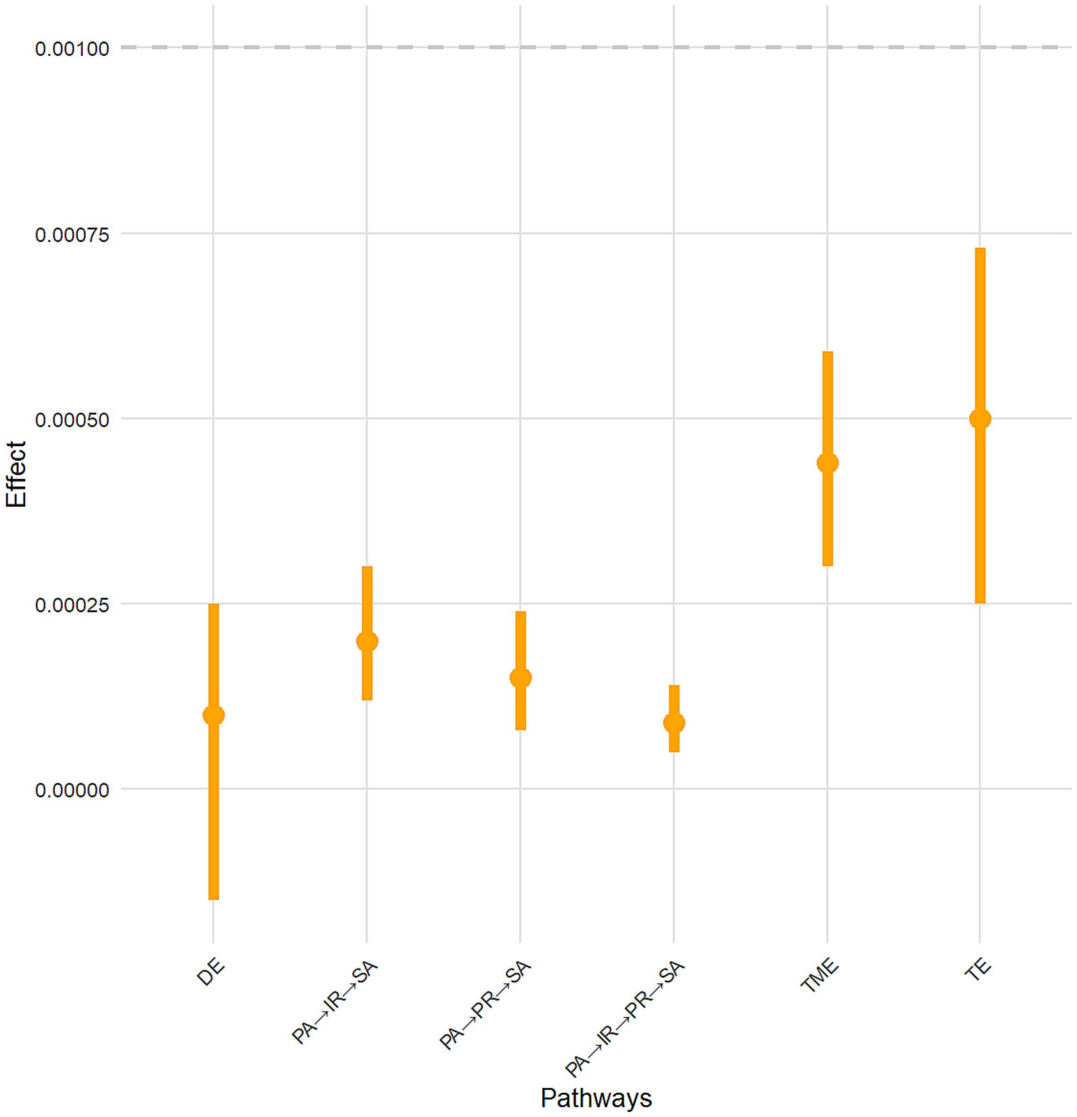
Visualization graph of mediating effect testing. DE, direct effect; TME, total mediating effect; TE, total effect. PA, physical activity; IR, interpersonal relationships; PR, psychological resilience; SA, social adaptation.

**Figure 4:**
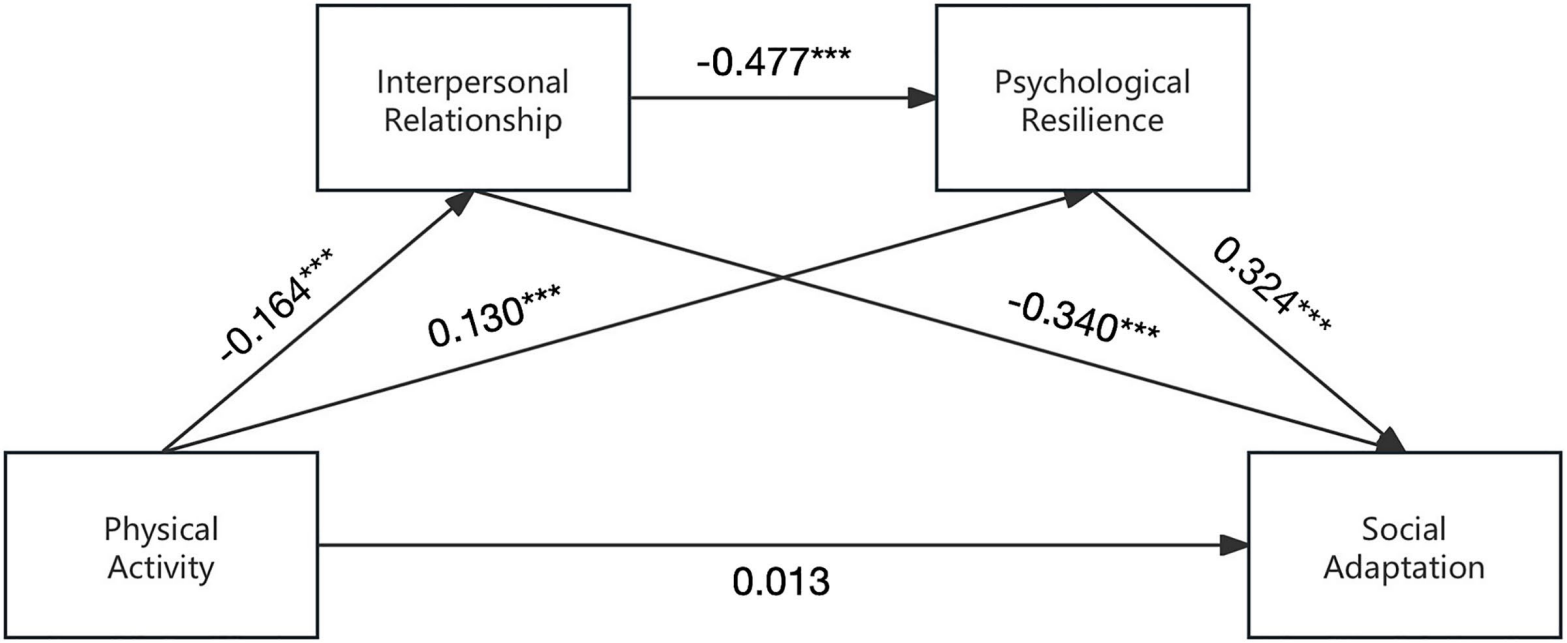
Chain mediating paths between physical activity and social adaptation. ***p < 0.001.

Further, using Model 6 in the SPSS plug-in program PROCESS3.5, the chain mediating effects of interpersonal relationships and psychological resilience as mediators in the association between physical activity and social adaptation was tested using 5,000 Bootstrap repeated sampling. When the 95% confidence interval did not include 0, the mediating effect was statistically significant. The results are shown in Table 5 and Figures 3, 4.

**Table 5:** Mediation effects of positive interpersonal relationships and psychological resilience in the association between physical activity and social adaptation.

Type of effect	Path	Effect value	SE	95% CI Lower limit	95% CI Upper limit	Proportion of variance
Direct effect	PA → SA	0.0001	0.0001	−0.0002	0.0002	20%
Indirect effect 1	PA → I*R* → SA	0.0002	0.0001	0.0001	0.0003	40%
Indirect effect 2	PA → P*R* → SA	0.0001	0.0000	0.0001	0.0002	20%
Indirect effect 3	PA → I*R* → P*R* → SA	0.0001	0.0000	0.0001	0.0002	20%
Total indirect effect		0.0004	0.0001	0.0003	0.0006	80%
Total effect		0.0005	0.0001	0.0003	0.0007	100%

SE, standard error; PA, physical activity; IR, interpersonal relationships; PR, psychological resilience; SA, social adaptation.

The direct effect of physical activity on social adaptation included 0 in the Bootstrap 95% confidence interval [−0.0002, 0.0002], indicating that the direct effect of physical activity on social adaptation was not significant. The total indirect effect of interpersonal relationship score and resilience as mediating variables did not include zero in the Bootstrap 95% confidence interval. This total indirect effect included three paths. The path of “physical activity → interpersonal relationships → social adaptation” had a significant mediating effect (standardized effect size = 0.0002, accounting for 40% of the total effect). The path of “physical activity → psychological resilience → social adaptation” was significant (standardized effect size = 0.0001, accounting for 20% of the total effect). The chain mediation path of “physical activity → interpersonal relationships → resilience → social adjustment” had a significant mediating effect (standardized effect size = 0.0001, accounting for 20% of the total effect).

## 4 Discussion

### 4.1 Relationship between physical activity and social adaptation

This study elucidated the relationship between physical activity and social adaptation among high school students. The results demonstrated a significant positive correlation between physical activity and social adaptation, confirming Hypothesis 1. This finding aligns with prior research (25, 63) and points to the possibility that physical activity is associated with enhanced adolescents’ social adaptation, consistent with our model. However, the direct predictive effect of physical activity on social adaptation became non-significant upon the simultaneous inclusion of the two mediating variables in the model. This suggests that any influence of physical activity on social adaptation is fully mediated by the two proposed mechanisms.

The unifying theory of physical activity postulates that physical activity influences social adaptation by enhancing improve emotional (64). A comparative analysis of physical education curriculum standards between China and the United States reveals that while both nations address social adaptation, the U.S. standards place greater emphasis on its cultivation, detailing specific social adaptive competencies expected at this educational stage (65). During high school, one can demonstrate responsible personal and social behavior in a physical activity environment (65). Students are thereby encouraged to leverage the social interaction opportunities inherent in many forms of physical activity and demonstrate appropriate social behaviors during these activities (66). physical activity help adolescents participate more in social activities and integrate into society (67). Interventions involving physical activity appear to influence individuals’ social competence, vitality, and physical strength (68). Physical activity, especially in group or team environments, essential for adjusting to social changes (42). Furthermore, regular engagement in physical activity bolsters students’ self-confidence and self-efficacy, which are crucial for deriving positive emotional experiences and happiness (69). Specifically, engaging in physical activity of sufficient duration and intensity may enhance the social adaptive abilities of high school students. Therefore, it is imperative to encourage high school students to incorporate regular physical activity into their daily routines to foster successful adaptation to modern society.

### 4.2 Positive interpersonal relationships mediate the relationship between physical activity and social adaptation

The results confirm that interpersonal relationships significantly mediate the association between physical activity and social adjustment among high school students, supporting Hypothesis 2. This finding is consistent with established literature (70, 71).

The higher the score of the interpersonal relationship scale used in this study, the lower the quality of interpersonal relationships; conversely, the lower the score, the higher the quality of interpersonal relationships. Therefore, engagement in physical activity may affect the development of interpersonal competencies, positive peer relationships, cooperation, and positive social interactions (72). Adolescents who regularly participate in structured physical activity may demonstrate enhanced communication skills (73). Since the physical activity of most high school students happens in group settings, physical activity inherently fosters cooperative behaviors (74) and may creates numerous opportunities for social interaction and communication (75). These activities thus represent an effective strategy for promoting positive peer engagement, while simultaneously reducing interpersonal distress (76). Within the school context, interpersonal relationship quality exert substantial influence on adolescents’ psychological well-being. The teacher-student relationship, can serve as a developmental resource that fosters positive belief systems and enhances social adaptation (77). Furthermore, constructive interpersonal relationships that develop through physical activity could strengthen teamwork capabilities and leadership skills (78). In the daily context of Chinese adolescents, moderate extracurricular physical activities (such as jogging, ball games, and swimming) can enhance the function of the prefrontal cortex, improve executive control and cognitive flexibility, and play a role in providing alternative sources of value and opportunities for reintegration into society, thereby influencing social adaptation (79). Furthermore, physical activities such as team sports and group exercise classes involve frequent involve social interaction. In these collaborate environments, individuals work with teammates toward shared goals, which fosters social engagement and may leads to positive social experiences (50).

As an integral component of daily life, physical activity may provide structured contexts for social engagement. Given humans’ fundamental social nature, participation in physical activity helps coordinate interpersonal dynamics, refine communication abilities, and develop comprehensive skills necessary for navigating social expectations (80). Adolescent social adaptation develops through practical experience, with physical activity serving as a crucial platform for generating positive interpersonal connections, thereby effectively influencing adolescent social adaptation (71).

### 4.3 Psychological resilience mediates the relationship between physical activity and social adaptation

The results suggest that psychological resilience is a significant mediator in the relationship between physical activity and social adaptation among high school students, thereby confirming Hypothesis 3. This finding aligns with previous empirical work (81).

Research indicates that regular, moderate-intensity physical activity enhances subjective well-being and fosters the development of positive psychological traits, including resilience (82). Adolescents with higher psychological resilience typically may possession protective resources such as optimism and persistence. When confronted with stressors, these students may be able to effectively mobilize and deploy these resources, enabling them to navigate challenges more successfully. Evidence has indicated a strong association between psychological resilience and social adaptation, where greater resilience correlates with more efficient utilization of coping resources and a stronger perceived sense of control over one’s environment (83). Low frustration experienced in different physical activity contexts (i.e., physical education and leisure-time physical activity) reports high well-being and enjoyment (84), which may have an impact on social adaptation. Studies have shown that short-term football training programs can have an impact on psychological resilience and further influence social adaptation (85). Within physical activity contexts, support systems—including material and emotional support from family, peers, and schools—constitute essential resources for fulfilling fundamental psychological needs. When these supportive conditions are present, individuals demonstrate greater propensity to develop positive characteristics and enhance their resilience capacity. This elevated resilience, in turn, strengthens their ability to mobilize various resources to confront adversity effectively, ultimately may improved social adaptation outcomes.

### 4.4 Chain mediation roles of interpersonal relationships and psychological resilience

The findings indicated that interpersonal relationship quality and psychological resilience function as sequential mediators in the relationship between physical activity and social adaptation among high school students, thereby confirming Hypothesis 4.

Analysis of the chain mediation pathway revealed that physical activity influences social adaptation through the sequential intermediaries of positive interpersonal relationships and psychological resilience. Specifically, physical activity appears to facilitate the development of positive interpersonal relationships among high school students, which in turn serve as protective factors that enhance psychological resilience. High school students with strengthened resilience demonstrate a greater capacity to withstand social pressures and adversities, because resilience was more “protective” against distress (86). Therefore, adolescents consequently achieving better social adaptation.

Within this chain mediation model, the direct path coefficient from physical activity to social adjustment was positive but statistically non-significant. The two mediating variables we tested—positive interpersonal relationships and psychological resilience—fully accounted for the association between physical activity and social adjustment, with the mediation effect attenuating the direct influence of physical activity on social adaptation (87).

The established mediation model suggests that physical activity may be an antecedent of social adaptation. However, positive interpersonal relationships and psychological resilience operated as sequential mediators that could further explicate the proposed impact of physical activity on social adaptation. This chain mediating mechanism points to a potential underlying process through which physical activity influences social adaptation in high school students, providing valuable practical implications for developing interventions aimed at enhancing their adaptive functioning.

## 5 Implications

This study validates the relationships between physical activity, positive interpersonal relationships psychological resilience, and social adaptation among high school students, and revealed the mediating mechanisms of interpersonal relationships and psychological resilience in the association between physical activity and social adjustment of senior high school students. The results expand current conceptualizations of the positive psychological effects of physical activity among senior high school students and deepen the understanding of these relationships, broadening the research perspective on factors influencing adolescent social adaptation.

This findings provide evidence and suggestions for optimizing high school students’ social adaptation. They highlight the need for society to prioritize adolescents’ development of social adaptation and create favorable conditions, offering a scientific basis for relevant policy-making. Encouraging collaborative efforts from society, schools, and families to build conducive physical activity environments can promote active student participation, encourage them to choose more team sports, optimize social adaptation, and lay a solid foundation for their adaptive development.

## 6 Limitations and future directions

This study has several limitations that should be addressed in future research. First, the cross-sectional design precludes the establishment of causal inferences. Future investigations would benefit from employing longitudinal approaches with expanded sample sizes to examine the underlying mechanisms across temporal dimensions and further elucidate causal pathways between variables. Second, the reliance on self-report measures of physical activity may introduce subjective response biases. Subsequent research could integrate more objective assessment tools, such as accelerometers, with advanced methodological approaches to enhance the validity of data collection. Finally, while this study examined mediation pathways using composite scores of the main constructs, future research should investigate the specific effects of individual subdimensions within each variable to provide a more nuanced understanding of the underlying mechanisms.

## 7 Conclusion

This study investigated the relationships among physical activity, interpersonal relationships, psychological resilience, and social adjustment among high school students. The results demonstrated that physical activity, interpersonal relationships, and psychological resilience all served as significant predictors of social adjustment. Furthermore, the constructed chain mediation model proved statistically significant, suggesting that physical activity may increase interpersonal relationships, which subsequently increase psychological resilience, ultimately fostering social adaptation.

By integrating social adaptation theory, self-efficacy theory, and the broaden-and-build theory of positive emotions, this research highlights the crucial roles of interpersonal relationships and psychological resilience in the relationship between physical activity and social adaptation. These findings broaden our understanding of the underlying mechanisms of social adaptation and provide insights for developing interventions to enhance social adjustment levels among high school students.

## Data Availability

The original contributions presented in the study are included in the article/supplementary material, further inquiries can be directed to the corresponding author/s.
